# Deficiency of microglial Hv1 channel is associated with activation of autophagic pathway and ROS production in LPC-induced demyelination mouse model

**DOI:** 10.1186/s12974-020-02020-y

**Published:** 2020-11-06

**Authors:** Man Chen, Lin-Lin Yang, Zi-Wei Hu, Chuan Qin, Luo-Qi Zhou, Ya-ling Duan, Dale B. Bosco, Long-Jun Wu, Ke-Bin Zhan, Sha-Bei Xu, Dai-Shi Tian

**Affiliations:** 1 Department of Neurology, Tongji Hospital, Tongji Medical College, Huazhong University of Science and Technology, Wuhan, 430030 Hubei People’s Republic of China; 2 Department of Neurology, Second Affiliated Hospital of University of South China, Hengyang, 421001 Hunan People’s Republic of China; 3 Department of Neurology, Mayo Clinic, Rochester, MN 55905 USA

**Keywords:** Demyelination, Microglia, Autophagy, ROS, Neuroinflammation

## Abstract

**Background:**

Multiple sclerosis (MS) is an immune-mediated demyelinated disease of the central nervous system. Activation of microglia is involved in the pathogenesis of myelin loss.

**Objective:**

This study is focused on the role of Hv1 in regulating demyelination and microglial activation through reactive oxygen species (ROS) production after lysophosphatidylcholine (LPC)-mediated demyelination. We also explored autophagy in this process.

**Methods:**

A model of demyelination using two-point LPC injection into the corpus callosum was established. LFB staining, immunofluorescence, Western blot, and electron microscopy were used to study the severity of demyelination. Microglial phenotype and autophagy were detected by immunofluorescence and Western blot. Morris water maze was used to test spatial learning and memory ability.

**Results:**

We have identified that LPC-mediated myelin damage was reduced by Hv1 deficiency. Furthermore, we found that ROS and autophagy of microglia increased in the demyelination region, which was also inhibited by Hv1 knockout.

**Conclusion:**

These results suggested that microglial Hv1 deficiency ameliorates demyelination through inhibition of ROS-mediated autophagy and microglial phenotypic transformation.

## Introduction

Multiple sclerosis (MS) is a chronic disorder of the central nervous system (CNS) that is characterized by pathological demyelination and inflammatory response [1]. Demyelination refers to loss of myelin around the axons, which leads to impaired transmission of nerve impulses, accompanied by neurodegeneration and inflammation. And it leads to neurological disorders with resultant impairment in spatial learning and memory [2, 3]. Inflammatory response is key to this demyelinating disease, affecting both the degree of myelin damage and remyelination. Early demyelinating and neurodegenerative changes in MS include microglia with pro-inflammatory phenotypes that express molecules involved in phagocytosis, oxidative damage, antigen presentation, and T cell co-stimulation [4].

Microglia are resident immune cells of the CNS which display a spectrum of activation states, which can be defined by a combination of their morphological and molecular phenotypes [4]. Following CNS injury, microglia may be broadly characterized as either pro-inflammatory, classically activated microglia (CAM), or as anti-inflammatory, alternatively activated microglia (AAM). CAM are characterized by the secretion of reactive oxygen species (ROS) and pro-inflammatory cytokines, whereas AAM are primarily concerned with anti-inflammatory functions [5]. Their range of functions, which are often opposing, plays a critical role in myelin repair. With the development of demyelinating disease, the transition of CAM to AAM in the lesion area contributed to OPC differentiation and initial remyelination [6, 7].

Autophagy is a conserved intracellular mechanism that maintains intracellular balance, in which damaged or dysfunctional proteins, lipids, and organelles are degraded by lysosomes [8]. Autophagy within microglia affects their inflammatory response by modulating reactive oxygen species (ROS) production. Autophagy scavenging of depolarized mitochondria reduced the overproduction of ROS, which had a protective effect in MS [9]. Our previous study had found that regulating autophagy within microglia reduced white matter damage [10]. However, there remain few studies on microglial autophagy in MS, and how autophagy affects microglial function is still largely unknown.

The novel voltage-gated proton channel Hv1, encodes by the gene Hvcn1, is highly expressed by immune system cells, and specifically by microglia in the CNS [11]. It was previously shown that Hv1 was necessary for microglia to produce nicotinamide adenine dinucleotide phosphate oxidase (NOX) dependent ROS and reduce NOX dependent acidification [12]. Recently, we have demonstrated that Hv1 deficiency shift microglia from CAM to AAM polarization state, reduced ROS, and pro-inflammatory cytokine secretion, thus alleviating brain injury after ischemic stroke [11, 13]. In addition, oxidative stress plays an important role in the pathogenesis of multiple sclerosis. In MS and its animal models, ROS is considered to be a mediator of demyelination and axon damage [14]. Free radicals are mainly produced by macrophages and are considered as the mediators of demyelination and axonal damage. Chronic inflammation increases ROS production. After that, antioxidant defense is damaged, leading to axonal demyelination and decreased or blocked conduction. In the immune response, activated microglia increased ROS, resulting in increased lipid peroxidation, while oligodendrocytes are more likely to be damaged by ROS. The degradation of myelin may be mediated by lipid peroxide. However, whether Hv1 affects microglial autophagy, thereby affecting local inflammatory response and MS-related white matter injury requires further study.

In this study, we found that Hv1 deficiency inhibited ROS production and modulated microglial function and phenotype. Moreover, Hv1 knock out could suppress microglial autophagic activation, which promoted myelin sheath repair following white matter injury. This suggested that Hv1-mediated autophagy regulates the local inflammatory microenvironment via ROS. Consequently, targeting Hv1 has therapeutic potential for modulating inflammation of CNS lesions, thereby promoting tissue repair and preventing disease progression in multiple sclerosis.

## Materials and methods

### Animals

All animal procedures were approved by the Institute of Animal Care Committee of Tongji Medical College, Huazhong University of Science and Technology, China. Mice were housed in the specific pathogen free (SPF) animal facility with water and food supplied ad libitum. They were kept in an alternating 12-h periods of light and dark cycle at the standard conditions of 22 °C temperature and relative humidity of 55–60%. Adult C57BL/6 male and female mice (wild type, WT; 20–25 g; 10-12 weeks old) were obtained from Hunan SJA Laboratory Animal Co. Ltd., Hunan, China. The murine strain Hv1^−/−^ (Hv1 knockout, Hv1 KO, Jackson Laboratory, Bar Harbor, ME, USA) was used wherever mentioned [13].

### LPC injection (two-point injection)

**Fig. 1:**
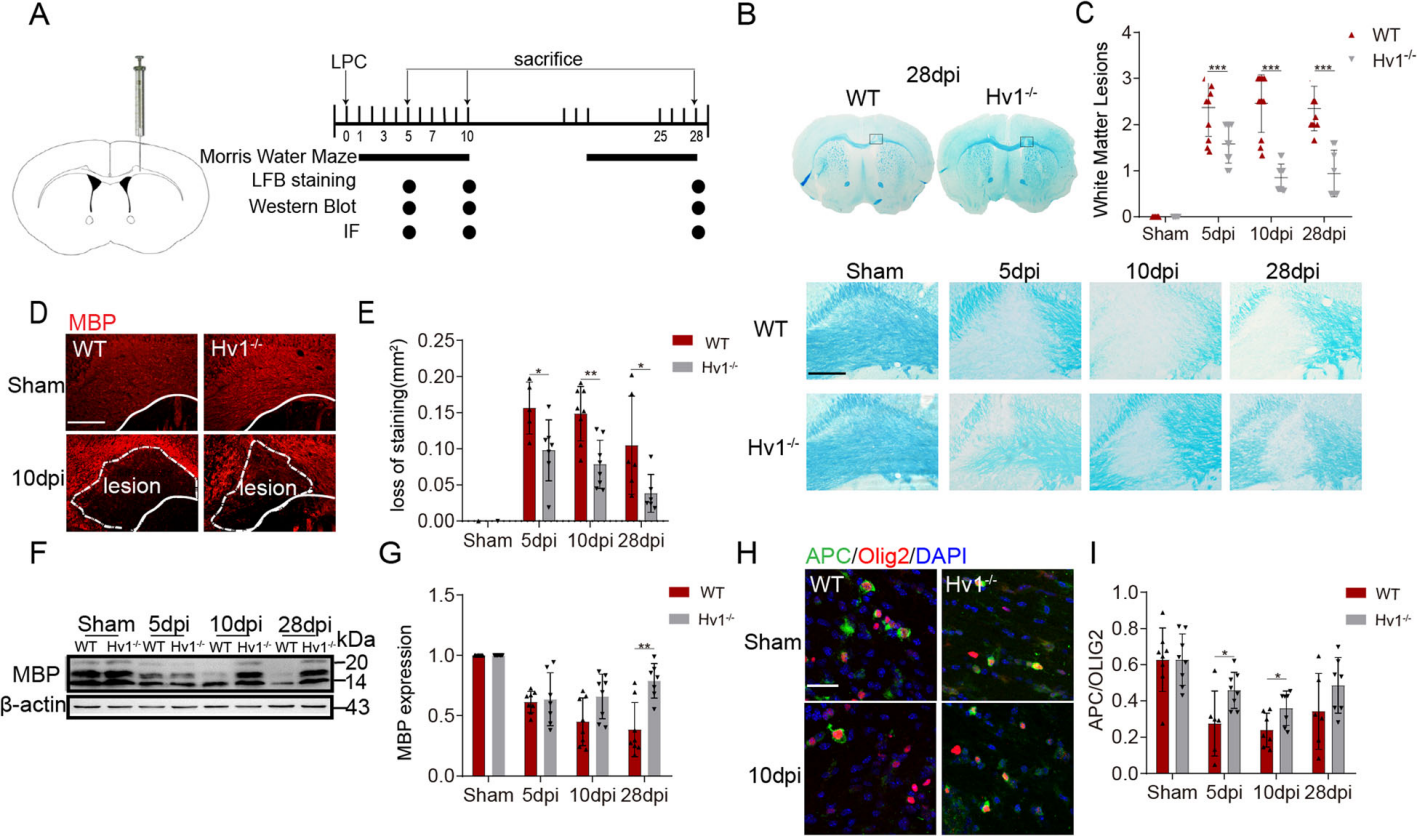
LPC-induced demyelination was ameliorated by Hv1 deficiency. **a** Illustration of injection positions (1.0 mm lateral, 0.6 mm anterior, 2.1 mm deep and 1.0 mm lateral, 1.1 mm anterior, 2.4 mm deep) and detection time points. **b** White matter lesions were detected by LFB staining at 5, 10, and 28 days post-injection (dpi) (scale bar, 200um). **c** Summarized data shows the severity of CC white matter lesions in histogram. White matter damage score: normal (grade 0). Disordered arrangement of nerve fibers (grade 1). Obvious cavitation formation (grade 2). Disappearance of myelin sheath fibers (grade 3). Two-way ANOVA with Dunnett’s post hoc test. ***P < 0.001, *N* ≥ 7 per group. **d** Representative images of MBP immunostaining in the CC of WT and Hv1^−/−^ mice. Lesion area outlined with a white dashed line (scale bar, 200 μm). **e** Quantification of the percentage of MBP loss area at three-time points. Each point of WT mice, *N* = 5-8 mice; Hv1^−/−^ mice, *N* = 6-8 mice. **f** Western blot analysis of MBP expression. **g** Quantification of the MBP intensity was measured and calculated as fold change over contralateral. *N* = 7-8 mice for each group. **h** Representative images of APC and Olig2 immunostaining in the CC of WT and Hv1^−/−^ mice (scale bar, 30 μm). **i** Quantification of the ratio of APC^+^/Olig2^+^. Each point of WT mice, *N* = 6-8 mice; Hv1^−/−^ mice, *N* = 7-8 mice. Data are shown as mean ± SD, *P < 0.05, **P < 0.01, two-way ANOVA with Dunnett’s post hoc test

Based on the research of Qianqian Luo et al. [15] with some modifications, mice were anesthetized with isoflurane (induced at 3%, and maintained at 1.2–1.6%), and positioned in a stereotaxic frame. Corpus callosum demyelination was induced by stereotaxic injection of 2 μL of 1% LPC (Sigma) in 0.9% NaCl solution at the rate of 0.5 μL/min using a 32-gage, two-inch needle attached to a 5 μl Hamilton syringe at two points. The first injection site was 1.0 mm lateral to the bregma, 1.1 mm anterior, and 2.4 mm deep. The second injection site was 1.0 mm lateral to the bregma, 0.6 mm anterior, and 2.1 mm deep. After injection, the needle was kept in each position for an additional 10 min. The day of injection was regarded as day 0. Mice were kept for a period of 5, 10, or 28 days (5 dpi, 10 dpi, and 28 dpi) and subsequently harvested for immunofluorescence staining or Western blot analysis (Fig. 1a).

### Morris water maze

Morris water maze (MWM) was performed according to the experiment of Charles V Vorhees [16] and was conducted to assess the spatial learning and memory abilities of the animals. The Morris water maze consisted of a white cylindrical tank (120 cm in diameter and 38 cm in height) and a hidden platform 10 cm in diameter, which submerged 1 cm below the surface of the water with a depth of 30 cm and a temperature of 25 °C. The pool was divided into four quadrants, and the platform was placed at the midpoint of a random quadrant. Each quadrant had a geometric pattern. Each mouse received seven consecutive days of training beginning on day 2 or day 20, then followed by 1 day of rest, and the test was conducted on day 10 or day 28. Before the first training, each mouse was placed on a platform for 15 s, and was allowed to swim freely for 30 s. Then it was assisted to rest on the platform for another 15 s. In each experiment, the mice were placed in four quadrants of water. The amount of time each mouse took to locate the hidden platform and the motion trail was recorded by digital camera and ANY-maze software (Stoelting, CO, USA). If it did not find the platform within 90 s, it was placed on the platform for 10 s at the end of the experiment, and the escape delay was recorded as 90 s. The platform was removed on day 10 or 28, and the animals were placed in the relative platform quadrant. The time each mouse spent was recorded in the target quadrant in 90 s. After the test, the animals were dried with a towel and kept warm.

### Preparation of brain samples

At 5, 10, and 28 dpi, mice were sacrificed under 5% isoflurane. For histologic staining and immunofluorescence, they underwent cardiac perfusion with 30 ml 0.1% phosphate buffer (PBS), followed by 30 ml 4% paraformaldehyde (PFA). PBS and PFA were precooled to 4 °C. After perfusion, the brain was removed, post-fixed in 4% PFA overnight (4 °C), and completely dehydrated in 30% sucrose. Serial 20 mm coronal sections were cut on a constant temperature (−20 °C) frozen slicer (1950 cm, Leica, Germany). For Western blot, the mice were perfused with ice-cold PBS containing 1 U/ml of heparin. The brain was extracted, rapidly frozen in liquid nitrogen-cooled isopentane, and stored at −80 °C for later use.

### Luxol fast blue stain

As previously described [17], histological changes to myelin of the corpus callosum were observed and graded by Luxol fast blue (LFB) staining. In brief, the brain slices were placed in LFB dye (G1030, Servicebio, Ltd., Wuhan, China) and heated at 60 °C for 6-8 h. After rinsing, the brain slices were differentiated alternately in a lithium carbonate solution and 70% ethanol, then dehydrated with 75%, 90%, and 100% ethanol. Finally, the slices were soaked in xylene for 5-10 min and sealed with neutral resin. Images were taken by with an optical microscope (DP 50, Olympus, Japan). White matter lesions were assessed in the medial part of the corpus callosum (CC) region. The severity of white matter lesions was classified as normal (grade 0), disordered arrangement of nerve fibers (grade 1), obvious cavitation formation (grade 2), and disappearance of myelin sheath fibers (grade 3). Data is presented as median ± quartile. Mann-Whitney *U* test was used for comparison between groups.

### Electron microscopy

Mice were anesthetized, and intracardiac perfusion was performed with 2.5% glutaraldehyde in 4% paraformaldehyde. The brain was removed and 1 mm thick coronal sections were cut. The corpus callosum was cut into 1 mm^3^ pieces, then post-fixed with 2.5% glutaraldehyde. The samples were observed under routine electron microscopy (Hitachi HT7700) at 200 kV, ×1700 magnification. The ratio of myelinated axon thickness to axon diameter (G-ratio) of 280 fibers (70 axons per mouse, 4 mice per group) was measured by Image J (National Institutes of Health, Bethesda, MD, USA). The G-ratio was directly proportional to axon diameter, which directly reflected the degree of myelin sheath formation around the axon. In addition, microglia under an electron microscope were defined by the following characteristics to determine the existence of autophagosome of microglia. The cytoplasm of microglia is electron-dense and the nucleus is lenticular, which can be distinguished from other types of cells. Microglia shows distinct heterochromatin pattern. There is a deep band of heterochromatin with high electron density near the nuclear envelope and a dense heterochromatin network in the whole nucleu s[18].

### Immunofluorescence and confocal imaging

For immunofluorescence staining, brain slices were permeabilized by 0.25% Triton-X100 in PBS, then blocked with 10% bovine serum albumin (Sigma–Aldrich) for 1 h at 37 °C. Slides were incubated with primary antibodies overnight at 4 °C and washed three times with PBS for 10 min. Slides were incubated with secondary antibody 1 h in the dark at room temperature. Then the sections were washed three times for 10 min each in PBS before taking pictures. Primary antibodies used for immunofluorescence were ionized calcium-binding adapter molecule1 (Iba-1,1:500,Wako Pure Chemical Industries); Fc RII/III receptor (CD16/32, 1:120, 553142, BD Pharmingen); macrophage mannose receptor (CD206, 1:100, AF2535, R&D system); myelin basic protein (MBP,1:200, 10458-1-AP, Proteintech); adenomatous polyposis coli (APC, 1:200, OP80, Millipore); cluster of differentiation 68 (CD68, 1:500, MCA1957, Bio-Rad); 8-hydroxyguanosine (8-OHG, 1:200, ab48508, Abcam); oligodendrocyte lineage transcription factor (Olig2, 1:200, 13999-1-AP, Proteintech); LC3B (1:200, A7198, ABclonal); P62/ SQSTM1(1:200, P0067, Sigma). Secondary antibodies labeled with FITC, AlexaFluor488, AlexaFluor594, or Cy3 were purchased from Jackson ImmunoResearch Laboratories. 4′,6-Diamidino-2-phenylindole (DAPI, 1 μg/ml, Thermo Fisher Scientific) was used for nuclear staining. Sections were imaged by confocal microscopy (FV1200, Olympus, Japan). Image J was used to analyze the resulting images. For immunoreactivity of 8-OHG, image J was used to analyze the average optical density of 8-OHG positive regions under the same conditions of immunofluorescence staining and the exposure intensity. Imaris was used to reconstruct microglia morphology in 3D [19, 20].

### Western blotting

The corpus callosum injury lesion of the mouse brain was extracted for Western blot analysis as previously described [21]. White matter tissue was then dissected with the RIPA buffer (Beyotime, China) containing PMSF and cocktail. Bovine serum albumin (BSA, Beyotime, China) was used as the standard for protein concentration determinations. Protein content in each sample (30 μg) was separated by sodium dodecyl sulfate-polyacrylamide gel electrophoresis (SDS-PAGE) of 8-12%. After electrophoresis, protein was transferred to NC membrane and incubated overnight with primary antibody at 4 °C. The membrane was then incubated with a secondary antibody for 1 h at room temperature. Images were collected using an Odyssey CLx Imager (LI-COR Biosciences). Finally, the integrated optical density (OD) of the signals was semi-quantified with Image J. β-actin was used as an internal control. Major Western blot antibodies included MBP (1:1000, 10458-1-AP; Proteintech); LC3B (1:1000, A7198, ABclonal); P62/SQSTM1(1:1000, P0067, Sigma); Beclin-1(1:1000, 11306-1-AP, Proteintech); β-actin (1:8000, 66009-1-lg, Proteintech). DelightTM 800 was combined with anti-mouse (H + L) and anti-rabbit (H + L) (1:4000, ABclonal).

### Statistical analysis

All values and error bars in the quantitative figures are expressed as mean ± SD except for LFB staining. SPSS 19.0 was used for statistical analysis. Significance was compared between groups by repeated analysis of two-factor analysis of variance (ANOVA), Mann-Whitney *U* test, or repeated measures of univariate analysis of variance. *P* < 0.05 was considered as statistically significant.

## Result

### LPC-induced demyelination was ameliorated by Hv1 deficiency

The demyelination of brain white matter induced by LPC is a classic model, which is limited to the injection area, accompanied by spontaneous myelination regeneration. It is a simple, stable, and easy to repeat demyelination model. LPC mainly leads to the cleavage of myelin membrane and the integrity of axon. As the immune response of demyelination induced by LPC is mild, most axons are not damaged [15, 22]. In this study, the demyelination lesion, caused by a stereoscopic injection of LPC, was manifested by partial myelin loss within the corpus callosum. LFB staining was used to evaluate the degree of myelin loss in WT and Hv1^−/−^ mice. In WT mice, obvious demyelination was observed at 5, 10, and 28 days after injection (dpi), indicating stable and persistent demyelination due to the two-point LPC injection. Compared with the WT group, Hv1^−/−^ mice showed less demyelination (Fig. 1b and c). In order to further assess the loss of myelin, myelin basic protein (MBP) was analyzed at each time point. We evaluated the area of MBP loss in the medial part of the CC. Obvious MBP loss was observed in the WT demyelination group. However, MBP loss in the Hv1^−/−^ demyelination group was reduced (Fig. 1d and e), in accordance with the above LFB staining. We also used Western blot to analyze MBP expression. In line with the immunofluorescence, Hv1 deficiency resulted in less MBP degradation (Fig. 1f and g).

APC and Olig2 are markers of mature oligodendrocytes and total oligodendrocytes lineage cells, respectively. Differentiation and maturation of oligodendrocytes is a key part of myelin sheath recovery following injury. The ratio of APC^+^/Olig2^+^ as determined by immunofluorescence could reflect the extent of oligodendrocytes maturation. In the WT group, the APC^+^/Olig2^+^ ratio was significantly reduced after LPC treatment. But in the Hv1^−/−^ group, the ratio was significantly higher (Fig. 1h and i). Therefore, these results suggested that the microglial Hv1 proton channel deficiency reduced LPC-mediated demyelination by promoting myelin repair.

### Microglial activation was attenuated after Hv1 deletion

**Fig. 2:**
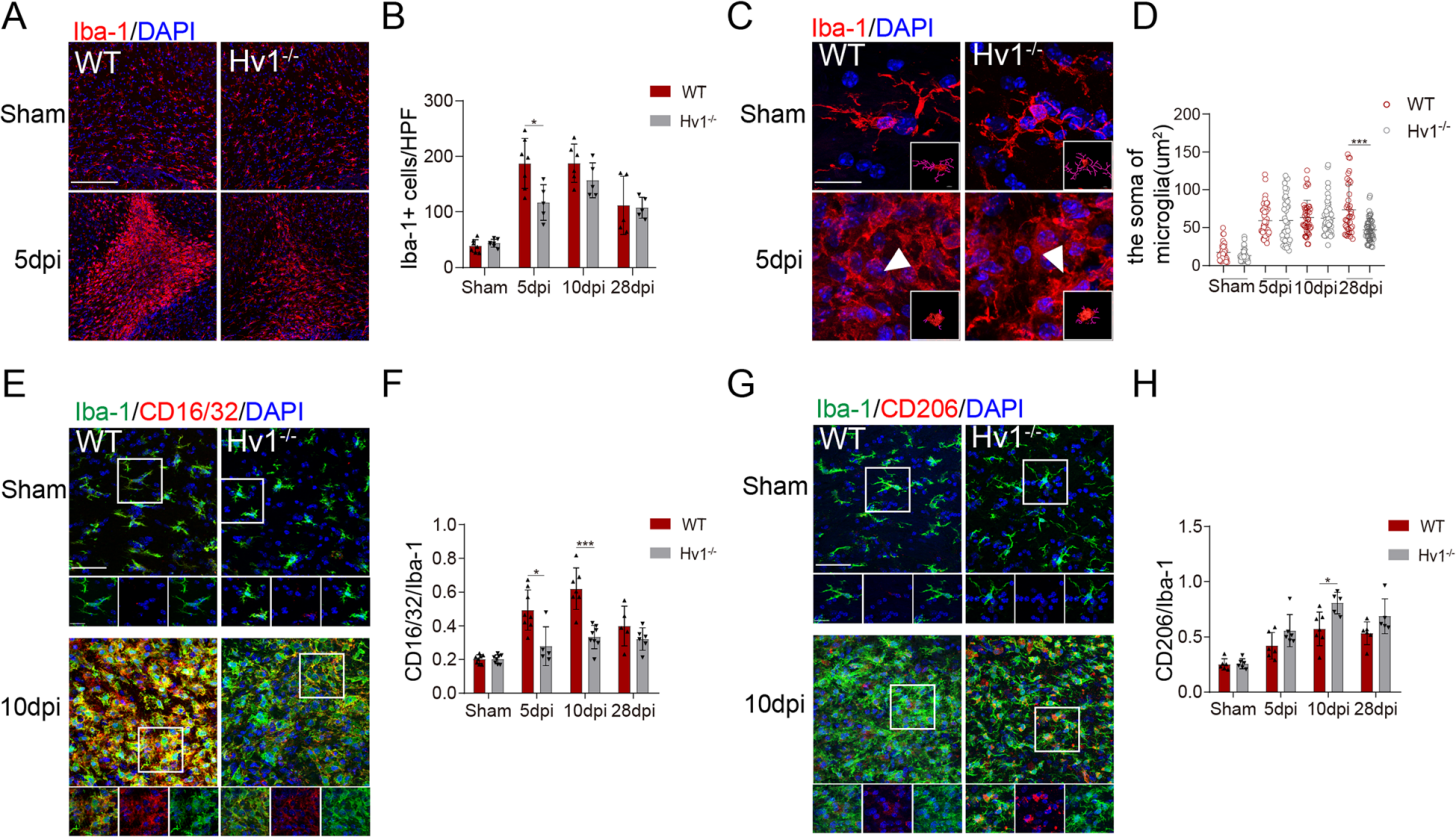
Microglial activation was attenuated by Hv1 deletion following LPC-induced demyelination. **a** Representative images of Iba-1 immunostaining in the CC of WT and Hv1^**−**/**−**^ mice (scale bar, 200 μm). **b** Quantification of the number of microglia per high-power field (HPF) in the CC. Each point of WT and Hv1^**−**/**−**^ mice, *N* = 5-7 mice. **c** Representative images of Iba-1 morphology and the corresponding 3D reconstructions (scale bars, magnified images, 20 μm; 3D reconstruction images, 5um) **d** Quantification analysis of the soma of microglia. Each point of WT and Hv1^**−**/**−**^ mice, *N* = 4-6 mice, 6-15 cells per mouse. **e** Representative images of Iba-1 and CD16/32 co-localization in the CC of WT and Hv1^**−**/**−**^ mice (scale bar, 50 μm; magnified images, 20 μm). **f** Quantification of the ratio of CD16/32^+^/Iba-1^+^. Each point of WT mice, *N* = 5-8 mice; Hv1^**−**/**−**^ mice, *N* = 5-8 mice. **g** Representative images of Iba-1 and CD206 co-localization in the CC of WT and Hv1^**−**/**−**^ mice (scale bar, 50 μm; magnified images, 20 μm). **h** Quantification of the ratio of CD206^+^/Iba-1^+^. Each point of WT mice, *N* = 6-7 mice; Hv1^**−**/**−**^ mice, *N* = 5-6 mice. Data are shown as mean ± SD, *P < 0.05, ***P<0.001, two-way ANOVA with Dunnett’s post hoc test

It has been shown that microglia turn toward a classically activated phenotype in MS [23]. Our previous studies have found that this pro-inflammatory microglial polarization exacerbates white matter injury [10]. As such, it was worth considering that the function of microglia and whether Hv1 can influence the activation of microglia in LPC-induced demyelination. Consequently, we next investigated microglial activation and morphological change in our model. Staining for myeloid cell marker ionized calcium-binding adapter molecule 1 (Iba-1) showed that microglia significantly aggregated in and around the CC injection area. However, in Hv1^−/−^ mice, there was considerably fewer aggregated microglia when compared to WT mice after LPC exposure (Fig. 2a and b). Morphological analysis of microglia in the injection area revealed a more ramified phenotype, with larger soma area in WT mice. Yet at the anaphase of demyelination (28 dpi), the soma of microglia Hv1^−/−^ mice were smaller than in WT mice (Fig. 2c and d). This suggests that Hv1 knockout inhibits microglial activation in the late stage of the disease.

Hv1 deletion has been shown to increase the number of anti-inflammatory microglia and attenuate brain damage following photothrombotic ischemic stroke [11]. Therefore, we investigated whether Hv1^−/−^ also has a protective role in demyelination. Fc RII/III receptor (CD16/32) and macrophage mannose receptor (CD206) are ubiquitous markers for classically activated microglia and alternatively activated microglia, respectively [24]. In WT demyelination mice, the percentage of CD16/32 and Iba-1 co-localization significantly increased compared to the WT sham mice. Meanwhile, a significantly smaller percentage was observed in the Hv1^−/−^ demyelination group (Fig. 2e and f). In parallel, CD206 showed an upward trend in immunofluorescence in Hv1^−/−^ mice, revealing that microglia transitioned toward a protective phenotype (Fig. 2g and h). Thus, these results indicate that Hv1 might facilitate the classical activation of microglia.

### Hv1 deletion reduced ROS production in the corpus callosum after LPC injection

**Fig. 3:**
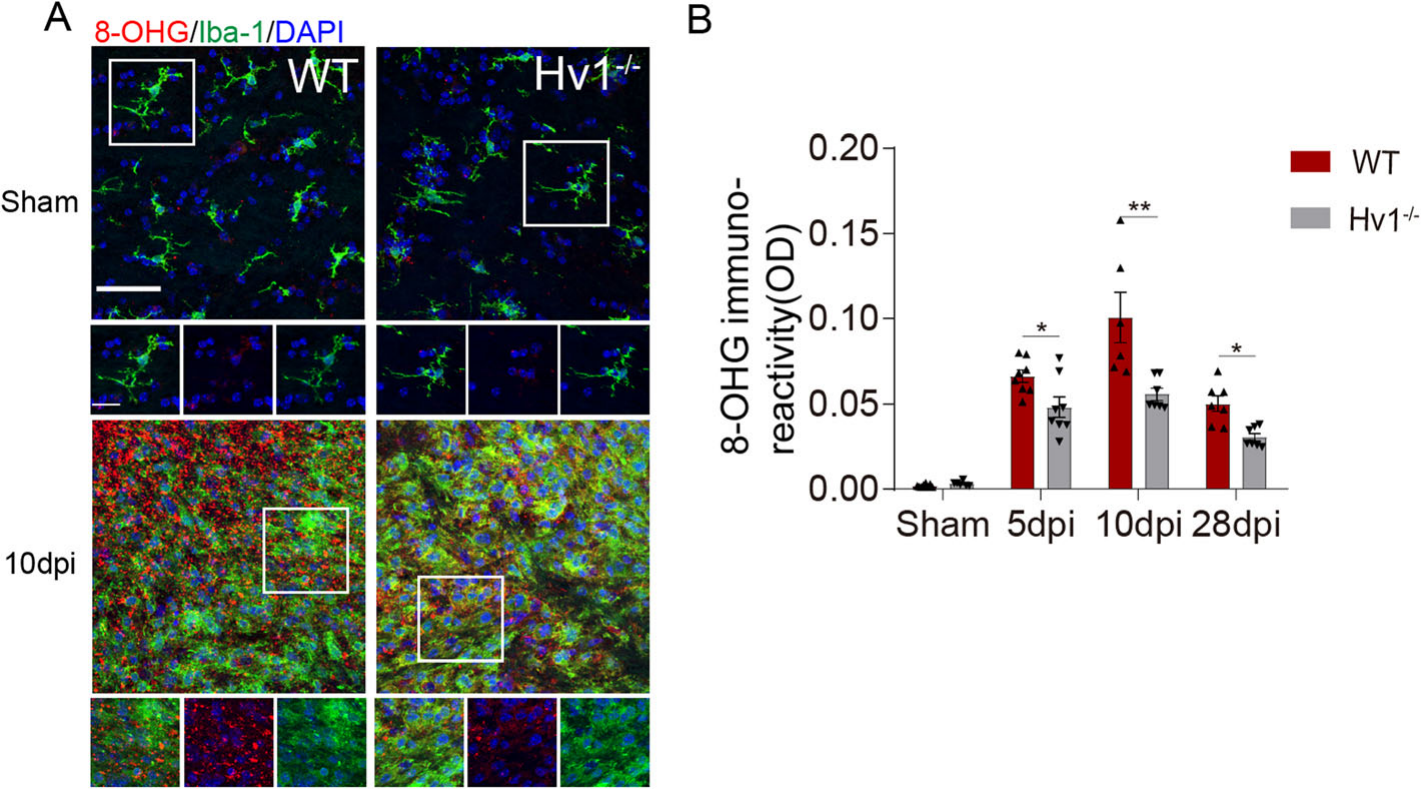
Hv1 deletion reduced ROS production in the corpus callosum after LPC injection. **a** Representative images of 8-OHG and Iba-1 immunostaining in the CC of WT and Hv1^**−**/**−**^ mice (scale bar, 50 μm; magnified images, 20 μm). **b** Quantification of the immunoreactivity of 8-OHG. Each point of WT mice, *N* = 6-8 mice; Hv1^−/−^ mice, *N* = 6-8 mice. Data are shown as mean ± SD, *P < 0.05, **P < 0.01, two-way ANOVA with Dunnett’s post hoc test

Hv1 is known to be involved in microglial NOX-dependent ROS production [13, 25]. Classically activated microglia promoted myelin breakdown via ROS and other substances that exacerbate MS, which manifests as increased demyelination and nerve injury [26]. Our previous studies have shown that Hv1 knockout attenuated microglia-derived ROS production in cuprizone-induced demyelination and ischemic stroke [11, 27]. We assumed that ROS played a role in microglia phenotypic transformation and autophagy activation. An oxidative stress marker, 8-hydroxyguanosine (8-OHG) was detected in the corpus callosum area. At 5, 10, and 28 dpi, we quantified the immunofluorescence intensity of 8-OHG, and it increased significantly compared with that in the WT sham group. Interestingly, the immunoreactivity of 8-OHG was substantially reduced in Hv1^−/−^ mice when compared with WT mice after LPC injection (Fig. 3a and b). Thus, these results indicate that microglial Hv1 deficiency suppressed ROS production in the corpus callosum after LPC injection.

### Hv1 knockout inhibited autophagosomes in microglia and facilitated myelinated ultrastructure after LPC-induced demyelination

**Fig. 4:**
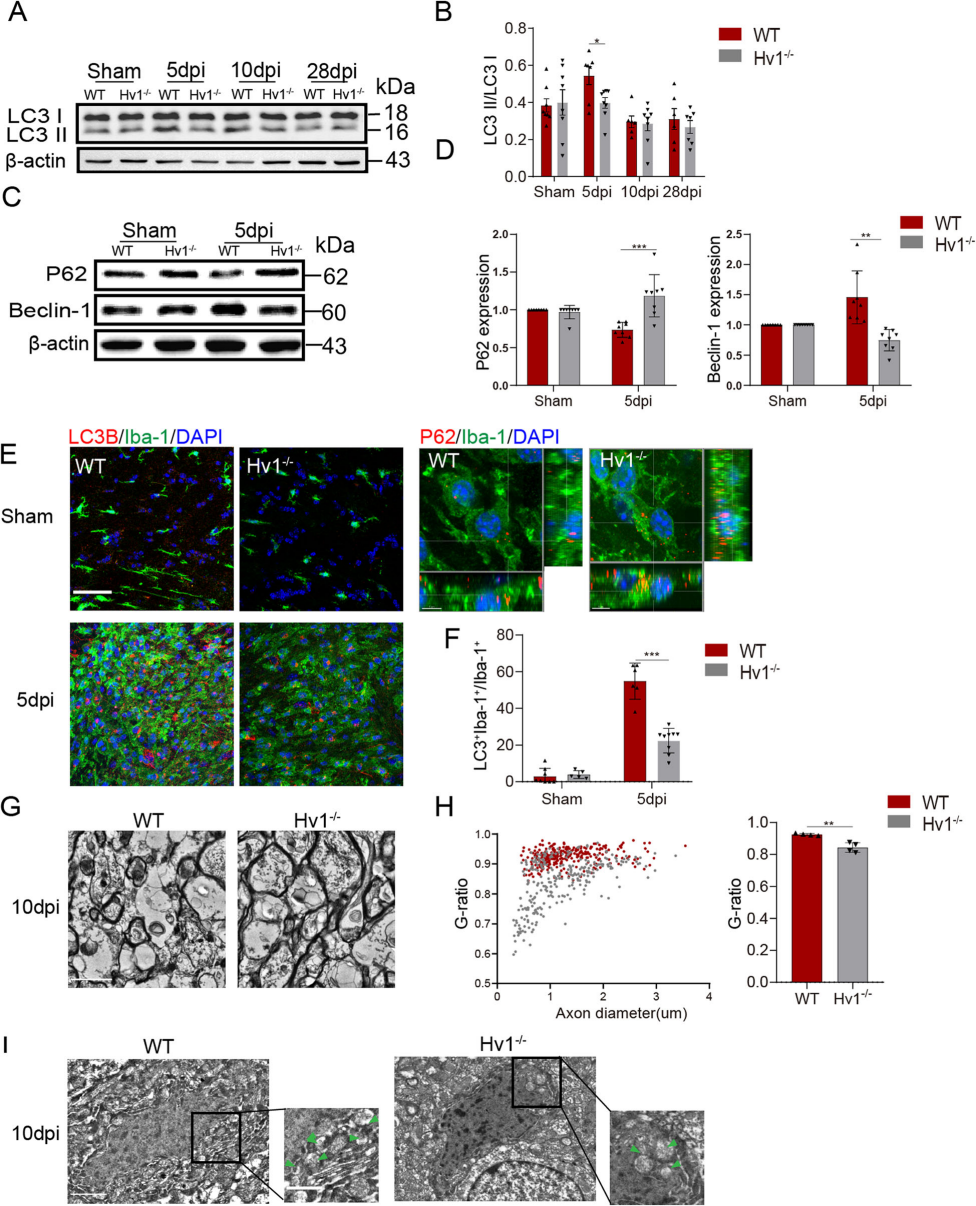
HV1 knockout inhibited autophagosomes in microglia and facilitated myelinated ultrastructure. **a** Western blot analysis of LC3 expression in the lesion of corpus callosum. **b** Quantification of the LC3 II/LC3 I was measured and calculated as fold change over contralateral. *N* = 6-8 mice for each group. **c** Western blot analysis of P62 and beclin-1 expression in the lesion of corpus callosum of 5 dpi. **d** Quantification of the P62 and beclin-1 was measured and calculated as fold change over contralateral. *N* = 8 mice for each group. **e** Representative images of Iba-1 and LC3B co-localization and P62 puncta in the CC of WT and Hv1^−/−^ mice of 5 dpi (scale bar, 50 μm and 5 μm). **f** Quantification analysis of the ratio of LC3B^+^Iba-1^+^/Iba-1^+^. WT mice of 5 dpi. *N* = 6-8 mice; Hv1^−/−^ mice, *N* = 5-9 mice. **g** Representative electron microscopy images for myelinated ultrastructure in the CC from different groups (scale bar, 2 μm). **h** Quantitative analysis of the ratio. *N* = 280 myelinated axons (70 axons per mouse, 4 mice per group) for each group. **i** Representative electron microscopy images showed the accumulation of autophagosomes in microglia in the CC at 10 dpi. Green arrows indicate autophagosomes (scale bar, 1 μm). Data are shown as mean ± SD, **P < 0.01, ***P < 0.001, two-way ANOVA with Dunnett’s post hoc test

Previous studies have shown that autophagy is involved in pro-inflammatory activation of microglia [10]. Additionally, accumulation of ROS was reported to induce autophagy [28, 29]. We hypothesized that Hv1 affects microglial autophagy via ROS inhibition. Microtubule-associated protein 1A/1B light chain 3 (LC3) is a soluble protein, which is widely distributed in mammalian tissues and cultured cells. At the same time, a cytoplasmic form of LC3 (LC3-I) binds to phosphatidylethanolamine to form a LC3-phosphatidylethanolamine conjugate (LC3-II), which is recruited into the autophagosome membrane. The flip of the autophagosome marker LC3-I to LC3-II reflects the increased autophagy activity [30, 31]. Furthermore, poly-ubiquitin-binding protein sequestosome 1 (SQSTM1, P62) tends to accumulate when autophagy is inhibited and to decrease when autophagy is induced. Monitoring LC3 and P62 levels can be used as markers of autophagy [32–34]. Beclin-1 also plays a key role in autophagy, helping to initiate autophagy [35]. Western blot analysis revealed that LPC treatment increased the expression of LC3 II and beclin-1, and downregulated p62 expression in the CC of WT. Hv1 knockout suppressed LPC-induced conversion of LC3 I to LC3 II, inhibited beclin-1 upregulation and increased p62 protein expression (Fig. 4a-d). Moreover, we also observed that Hv1 deficiency inhibited the upregulation of LC3 and iba1 double-positive microglia after LPC injection and increased P62 puncta (Fig. 4e and f). This indicates that LPC-induced demyelination could activate autophagy in microglia and Hv1 deletion suppressed this process.

Based on the above results, we next explored the ultrastructural changes of the myelin sheath at the peak of inflammation. Consistently, we found that destruction of myelinated structures was detected in WT demyelination mice, presenting as cavitation and delamination, while intact layered myelin sheaths were observed in the WT sham group. We used a quantitative analysis of the G-ratio to confirm the damage of myelin sheath ultrastructure. G-ratio was significantly reduced in Hv1^−/−^ demyelination mice compared with that of WT mice, suggesting that Hv1 deletion reduced myelin damage (Fig. 4g and h). In addition, electron microscopy showed that the formation of autophagic vesicles in microglia increased after LPC injection and decreased in the Hv1^−/−^ demyelination group (Fig. 4i). Therefore, we hypothesize that the decrease in autophagy in Hv1^−/−^ mice microglia might be related to the reduction of ROS.

### Hv1 deficiency rescued the spatial memory impairment of in LPC demyelinated mice

**Fig. 5:**
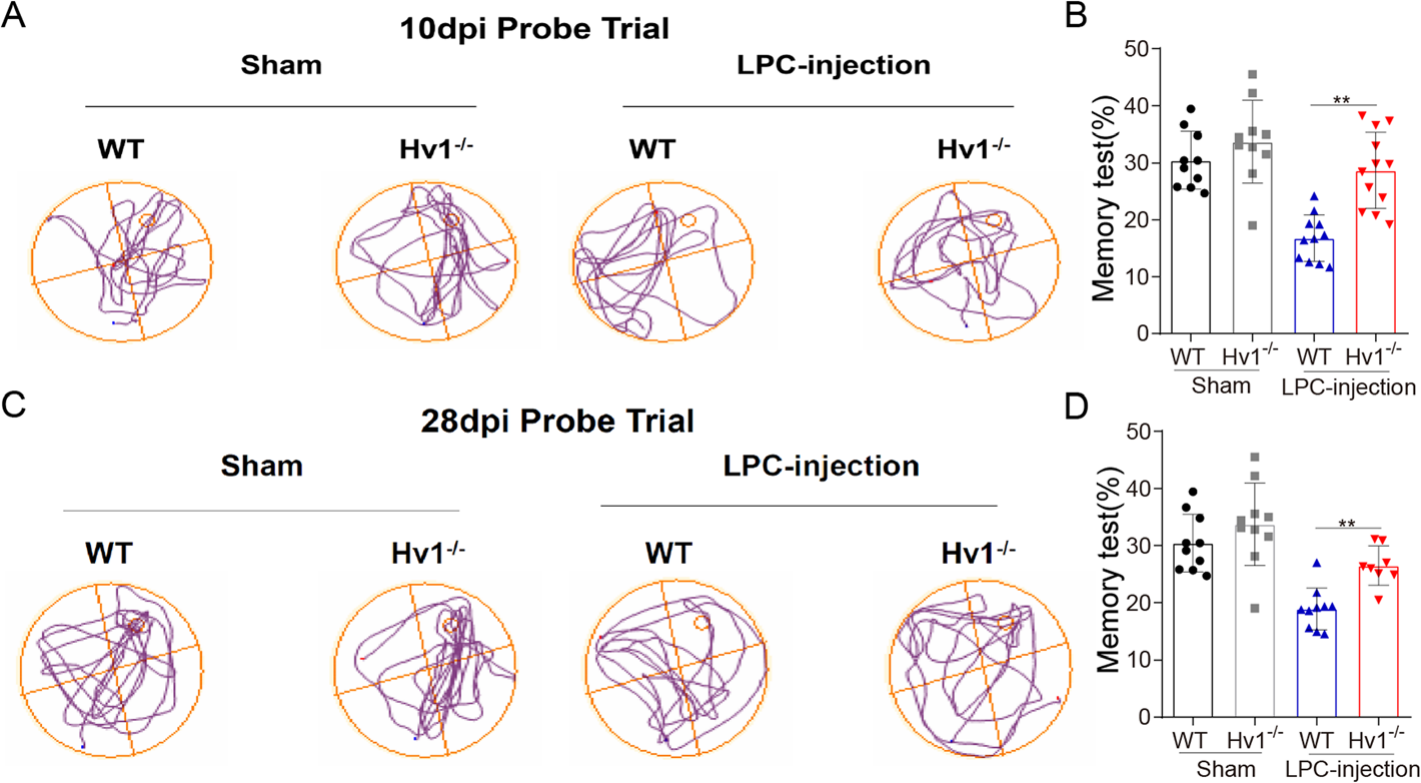
Hv1 deficiency rescued the spatial memory impairment induced by LPC treatment. (**a** and **c**) Representative motion trail of memory phase of WT and Hv1^−/−^ mice at 10 dpi and 28 dpi. (**b** and **d**) Quantification of the Morris water maze test at 10 dpi and 28 dpi of WT and Hv1^−/−^ mice, compared with the sham group. Each point of WT mice, *N* = 10-11 mice; Hv1^−/−^ mice, *N* = 8-12 mice. Data are shown as mean ± SD, **P < 0.01, two-way ANOVA with Dunnett’s post hoc test

To analyze the spatial memory ability of LPC injection in WT and Hv1^−/−^ mice, we monitored Morris water maze performance at 10 and 28 dpi. Consistent with previous studies [15], we found that spatial memory was significantly impaired in demyelination mice, as represented by the percentage of time spent swimming in the target quadrant, which decreased when the platform was absent. However, Hv1^−/−^ mice displayed remarkable improvement in the memory test (Fig. 5a-d). Overall, these results indicate Hv1 deficiency rescued the spatial memory impairment.

## Discussion

Microglia activation is an important feature of neuroinflammation and neurodegenerative diseases and can have both beneficial and adverse effects [4]. In multiple sclerosis, demyelination correlates with unresolved microglia activation and progressive inflammation [27]. It has been demonstrated that CAM lead to the release of pro-inflammatory cytokines that contributed to CNS damage. However, in late stages of MS, AAM dominate in the lesion and promote the resolution of inflammation by releasing anti-inflammatory cytokines [36]. Therefore, the study of factors affecting the function and state of microglia, and how we can transform CAM into AAM during disease progression can greatly increase control of diseases. In this study, we showed that the absence of Hv1 could facilitate the adoption of the AAM phenotype by microglia over the CAM phenotype and reduce ROS production, which improved demyelination to a certain extent. Interestingly, autophagy was observed to be involved in microglial phenotypic transformation and ROS production.

In demyelinating disease, microglia play a vital role. The proliferation and activation of microglia are the factors of demyelinating injury. But microglia can engulf apoptotic cells and myelin fragments, which helps myelin sheath regeneration and affects the maturation of oligodendrocytes [37, 38]. Many studies have shown that regulating the functional phenotype of microglia had an obvious protective effect on a variety of CNS diseases, such as neurodegenerative diseases [39], stroke [11, 13, 40, 41], intracerebral hemorrhage [42], and white matter ischemic disease [10]. In this study, our results showed that regulating the functional phenotype of microglia rather than merely inhibiting microglia, had a definite protective effect in demyelinating disease.

Hv1 is specifically expressed in microglia in the central nervous system. Hv1 is a proton channel that mainly helps NADPH oxidase produce reactive oxygen species by exhaling intracellular protons. Our previous study found that Hv1 deficiency had a protective effect following ischemic stroke [11]. The present study found that Hv1 knockout had a beneficial effect on LPC-induced demyelination. But the specific role of Hv1 in disease is not conclusively understood. It has been reported that microglial Hv1 promotes cuprizone-induced demyelination through oxidative damage [27]. Significant ROS production was also observed in the corpus callosum after LPC injection. ROS are important signaling molecules in oxidative stress. Under pathological conditions such as traumatic brain injury, ischemia/reperfusion, and hypoxia, the relatively excessive accumulation of ROS can destroy intracellular homeostasis, leading to oxidative stress and mitochondrial dysfunction [43]. In our study, ROS production was significantly reduced by Hv1 knockout.

**Fig. 6:**
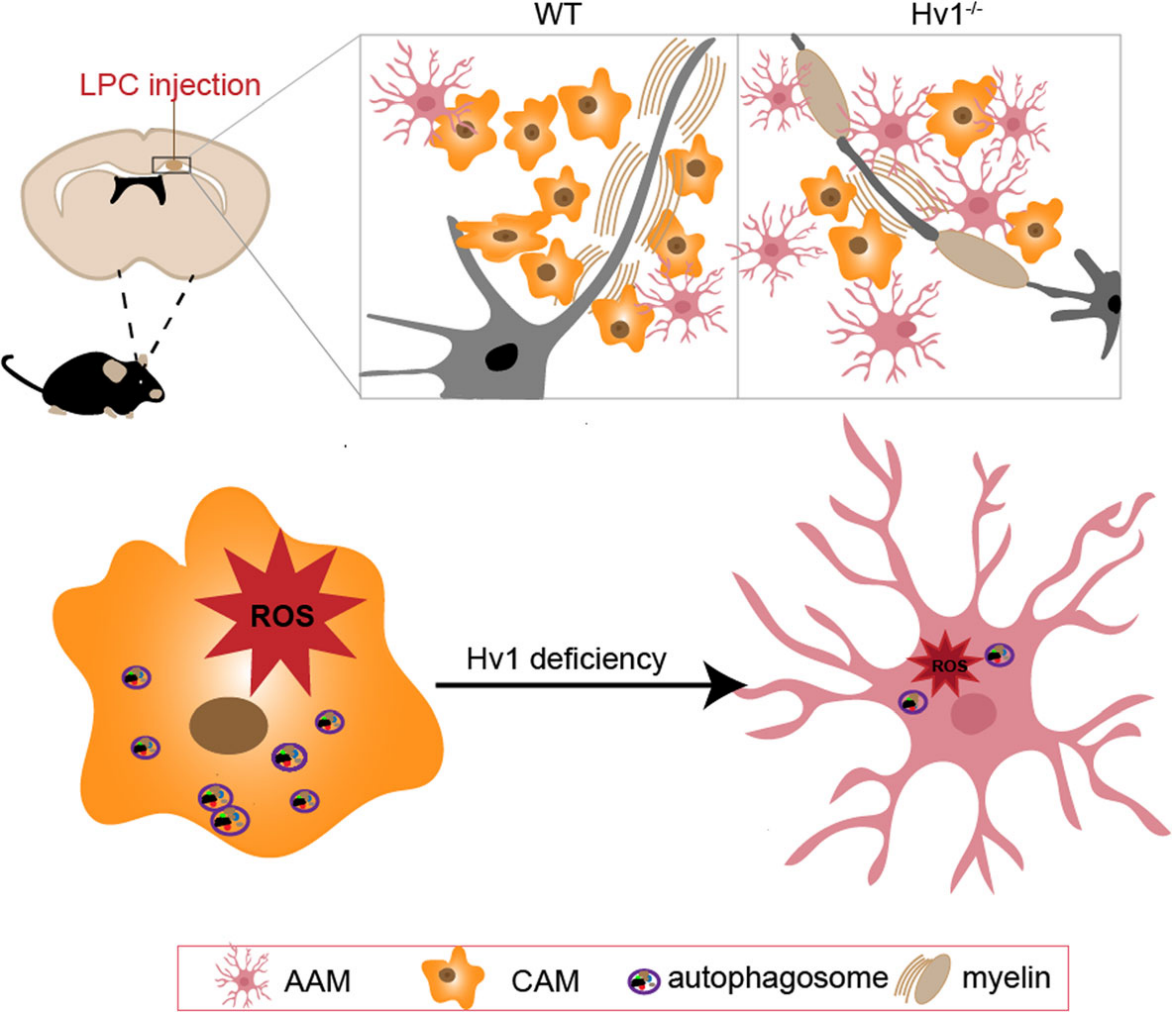
A summary figure which showed that microglial Hv1 channel activates autophagic pathway and promotes LPC-induced demyelination through ROS production

The autophagic pathway was activated in order to reduce damage due to ROS [44–46]. Autophagy is a core regulator of aging and neurodegeneration in the central nervous system. Transmission of toxic molecules and organelles to lysosomes via autophagy is essential for the health and survival of neurons. Microglial autophagy regulates phagocytosis and inflammation [47]. It has been reported that autophagy was conducive to synaptic homeostasis and confers resistance to environmentally induced oxidative stress [29, 48], but it has also been reported that autophagy induces microglial activation and has a negative effect on ischemic white matter damage and intracerebral hemorrhage [10, 49]. Importantly, we determined autophagy was inhibited in Hv1^−/−^ mice. Additionally, Hv1 deficiency was shown to reduce ROS. Therefore, we speculate that autophagy is related to ROS, and the protective effect of Hv1 deficiency might be due to ROS-mediated autophagy inhibition (Fig. 6).

## Conclusion

In conclusion, our study showed a relation between ROS, autophagy, and microglia. In the process of LPC-induced demyelination of corpus callosum, microglia produced cytotoxic mediators, such as ROS, leading to severe loss of local myelin sheath and a reduction of mature oligodendrocytes. It was accompanied by microglia adopting a classically activated phenotype and autophagy overactivation. We found that the loss of Hv1 in microglia could alleviate the damage caused by LPC to myelin sheath, which might be via decreasing ROS production and inhibiting ROS-mediated autophagy. Therefore, our findings, starting from ROS and autophagy, provide a promising therapeutic target for the treatment of MS. However, further research into the activation of autophagy after Hv1 deficiency is needed to elucidate details concerning the relationship between Hv1 and autophagy in microglia. And whether enhancing or inhibiting ROS can alleviate demyelination damage also needs further exploration.

## Data Availability

Not applicable.

## Abbreviations

MS: Multiple sclerosis
ROS: Reactive oxygen species
LPC: Lysophosphatidylcholine
LFB: Luxol fast blue
CNS: Central nervous system
CAM: Classically activated microglia
AAM: Alternatively activated microglia
NOX: Nicotinamide adenine dinucleotide phosphate oxidase
SPF: Specific pathogen free
MWM: Morris water maze
PBS: Phosphate buffer saline
PFA: Paraformaldehyde
CC: Corpus callosum
Iba-1: Ionized calcium-binding adapter molecule1
MBP: Myelin basic protein
APC: Adenomatous polyposis coli
CD68: Cluster of differentiation 68
8-OHG: 8-hydroxyguanosine
OD: Optical density
dpi: Days after injection
